# Comparison of dietary share of ultra-processed foods assessed with a FFQ against a 24-h dietary recall in adults: results from KNHANES 2016

**DOI:** 10.1017/S1368980022000179

**Published:** 2022-05

**Authors:** Sukyoung Jung, Sohyun Park, Jee Young Kim

**Affiliations:** 1 Department of Epidemiology, Milken Institute School of Public Health, George Washington University, Washington, DC, USA; 2 Department of Food Science and Nutrition, Hallym University, 24252 Chuncheon-si, South Korea; 3 The Korean Institute of Nutrition, Hallym University, Chuncheon-si, South Korea; 4 National Food Safety Information Service, Seoul, South Korea

**Keywords:** Relative validity, 24-h dietary recall, FFQ, Ultra-processed foods, Korea National Health and Nutrition Examination Survey

## Abstract

**Objective:**

To evaluate the performance of an FFQ for estimating dietary contributions of NOVA groups to individuals’ diets with a specific focus on ultra-processed foods (UPF) compared with a single 24-h dietary recall (24HR).

**Design:**

All consumed foods and beverages assess with both a 109-item FFQ and a single 24HR were classified into one of four NOVA groups: natural or minimally processed foods (MPF), processed culinary ingredients (PCI), processed foods (PF) and UPF. The contributions of each NOVA group to daily intakes of energy, protein, fat, saturated fat, carbohydrate, fibre and Na were expressed as crude intake, energy-adjusted intake and percentage intake. Mean differences, correlation coefficients and joint classification were calculated for intakes of energy and nutrients from each NOVA group between the FFQ and the 24HR.

**Setting:**

The Korea National Health and Nutrition Examination Survey 2016.

**Participants:**

Adults aged 19–64 years (*n* 3189).

**Results:**

The smallest group-mean differences between the two methods were observed in UPF (2–40 %). The greatest average Pearson’s correlation coefficients between the FFQ and 24HR were shown in dietary contributions of UPF (*r* = 0·22–0·25 for MPF; *r* = 0·02–0·05 for PCI; *r* = 0·11–0·18 for PF; *r* = 0·26–0·30 for UPF). The greatest agreement in quartile classification between the FFQ and the 24HR was observed in dietary contributions of UPF (70·0–71·5 % for MPF; 64·2–68·8 % for PCI; 66·9–69·2 % for PF; 71·8–73·9 % for UPF).

**Conclusions:**

The use of the FFQ for estimating absolute intake of UPF may not be encouraged in its current form, but it still may be used for relative comparisons such as quantile categorisation.

Ultra-processed foods (UPF), which are foods and beverages with a high energy density and low nutritional quality, have become more dominant in the global diet over the past two decades^(1–5)^. The most prominent method of food classification based on food processing levels is the NOVA classification, which has four classes of foods and beverages: natural or minimally processed foods (MPF), processed culinary ingredients (PCI), processed foods (PF) and UPF^(2)^. In NOVA classification, UPF are defined as ‘*formulations of food substances often modified by chemical processes and then assembled into ready-to-consume hyper-palatable food and drink products using flavours, colours, emulsifiers and a myriad of other cosmetic additives*’^(6)^. Collective evidence exists that shows higher UPF consumption is linked with a 39–102 % increased risk of metabolic syndrome, high waist circumference (WC), low HDL-cholesterol levels and overweight/obesity from cross-sectional studies^(7–9)^ and with a 1·20- to 1·34-fold greater risk of depression, cardiovascular diseases, cerebrovascular diseases and mortality from prospective cohort studies^(8,9)^. As evidence is emerging, efforts to shift towards limiting UPF consumption have become a relevant and timely topic.

Observational epidemiologic studies, especially prospective cohort studies, have merit in unravelling the association between dietary intake and health outcomes^(10)^. Prior prospective studies that have examined the association of UPF consumption with disease outcomes among adults have used 24-h dietary recalls (24HR), diet/food records (DR) or FFQ as the main dietary assessment methods. In general, multiple 24HR or DR over a long period have been appreciated as the more appropriate way to estimate the usual intake of participants and to examine the association between diet and health outcomes than other methods, including FFQ; however, they are expensive and time-consuming^(10,11)^. Although FFQ have been administered as the most practical method in large-scale epidemiological settings since the 1990’s^(10–12)^, inevitable measurement errors would have attenuated measures of association even if measurement errors occurred randomly in most circumstances^(10,13,14)^. Specific to UPF consumption, several issues related to the use of the FFQ have been raised. First, FFQ that have been used in previous studies may not cover the full spectrum of UPF consumption due to the limited number of predefined food lists and the lack of information on ingredients, cooking methods, eating place and the brand names of the packaged foods^(15)^. Second, although the FFQ were validated at the time of their inception as appropriate, they were not developed and/or validated in the explicit scope of UPF classification and/or their association with health outcomes. These concerns of FFQ use in UPF classification have become more evident in Korea because traditional Korean diets mainly consist of many dishes with combinations of individual ingredients and seasoning, which cannot be accurately classified using the food-based FFQ^(11)^.

In Korea, UPF consumption gradually increased between 2010 and 2018^(5)^ in parallel with the global trend; however, UPF and disease outcome associations have rarely been investigated. To date, no prospective study is available, and only one cross-sectional study has been conducted under the Korean scope, indicating the positive association between UPF and obesity in Korean women^(16)^. Since this may be due to the difficulty in the classification of foods assessed with the FFQ in Korean prospective cohort studies, the evaluation of the FFQ’s performance in UPF classification would provide new insights for future use of the FFQ.

With a nationally representative sample of Korean adults, we aimed to evaluate the performance of the FFQ to classify UPF consumption of individuals’ diets compared with the 24HR and examine the possibility of using FFQ in large-cohort studies that only measure dietary intake with FFQ.

## Methods

### Study population

The Korea National Health and Nutrition Examination Survey (KNHANES), an ongoing, cross-sectional and nationally representative survey, was established to monitor the health and nutritional status of the non-institutionalised civilian population in Korea^(17)^. The KNHANES has been conducted by the Korea Disease Control and Prevention Agency since 1998 and used a complex and multistage probability sampling design for representativeness of the non-institutionalised Korean population^(17)^. Since 2016, the KNHANES sample was selected from 192 primary sampling units (PSU), which are approximately 200 000 small geographical areas covering the whole country^(18)^. Each PSU consisted of sixty households and twenty-three target households were selected using systematic sampling^(18)^. All individuals aged ≥ 1 year within the selected households were considered eligible for further participation in the KNHNAES^(18)^. In 2016, a total of 10 806 persons were screened and asked to participate in the KNHANES. Among them, 8150 persons completed at least one or more surveys among the three-component surveys (health interview, health examinations, and nutrition surveys), and thus response rates were 75·4 %^(18)^. The present study was based on participants aged 19 years and older who completed the nutrition survey in 2016. More details of the KNHANES are available elsewhere^(17,18)^.

In the current analysis, we considered the FFQ as the surrogate measure and the 24HR as the reference method. Among 4750 participants aged 19–64 years, individuals were excluded if they had the following conditions: missing weights for health interviews and nutrition surveys (*n* 947), incomplete information on the 24HR or the FFQ (*n* 545) and extreme FFQ-assessed energy intakes (< 800 kcal or > 4000 kcal for men, < 500 kcal or > 3500 kcal for women) (*n* 69). The final analysis sample included 3189 participants (1215 men and 1974 women) (see online supplementary material, Supplemental Fig. 1).

### Dietary assessment

For the nutrition survey in the KNHANES, trained dieticians comprehensively collected dietary data, including two dietary assessment methods (1-day 24HR and FFQ). Eight dieticians were grouped into teams of four and conducted in-person interviews in participants’ homes using the computer-assisted personal interviewing system. Dietary information was collected 1 week after the health interviews and examinations.

#### FFQ

For the participants aged 19–64 years, the dish-based semiquantitative FFQ, newly developed in 2009^(19)^, was administered to collect information on the intakes of 112 food/dish items. The validity and reproducibility of the FFQ have been examined in detail elsewhere (reproducibility: correlation coefficients between the first and second FFQ, 0·54–0·61 (mean = 0·54) for nutrients, 0·33–0·87 (mean = 0·57) for food groups; validity: correlation coefficients between the first FFQ and the 12-day dietary records 0·29–0·45 (mean = 0·40) for nutrients)^(20)^. Participants reported how frequently they consumed the average portion sizes of 112 food/dish items over the preceding year. For the frequency of consumption, nine categories were generally available (never or less than once per month, once per month, 2–3 times per month, once per week, 2–4 times per week, 5–6 times per week, once per day, 2 times per day and 3 times per day), and as exceptions, milk items additionally asked types of milk (whole, low fat or similar); fruit items first asked if consumed seasonally or not and then frequencies accordingly; coffee items required further response if the respondent drank coffee more than 3 times per day. For the amount of consumption, three serving sizes (0·5, 1·0, or 1·5–2 times of standard) were available, and as exceptions, cooked rice, cooked mixed rice and kimbab (Korean rolls with dried seaweed) provided four serving size options because Koreans consumed those items as main dishes on a daily basis (0·5, 1·0, 1·5 or 2 times of standard); open-ended questions collected the amount of alcoholic beverages (soju, beer, makguli) if they consumed more than the greatest value of the given choice (more than 180 ml of soju, 200 ml of beer or 210 ml of makguli). In the NOVA system, alcoholic beverages can be classified as PF or UPF according to manufacturing procedures if those are considered foods^(21)^. Since alcohol is ‘*a toxic and psychoactive substance with dependence producing properties*’ and any alcohol use is related to some health risks^(22)^, we excluded dietary intakes from alcoholic beverages. Thus, a 109-item was used in the analyses.

#### 24-hour dietary recall

For participants aged 1 year and older, a single 24HR instrument was administered to collect information on description, quantity, and time and place of eating for all foods and beverages that the participants had consumed during the past 24 h (from midnight to midnight) for each of the main meals and any other eating occasion. During all four seasons, the 24HR was administered on either weekdays or weekends. The respondents were provided the quantity of consumed foods and beverages in units of volume with the assistance of tools such as standard measuring tools and/or two-dimensional measuring guides. The person in charge of cooking was further provided unique recipes for the home-cooked dishes. The 24HR in the KNHANES used the multiple-pass approach to minimise respondent burden and to enhance complete and accurate food recall. A proxy person (parents or legal guardian) responded on behalf of children or participants who had difficulty reporting their diet. For this study, we only used the data from participants aged 19–64 years.

#### Energy and nutrients intake estimation

For the 24HR, daily intakes of energy and nutrients were estimated by multiplying the intakes of all foods and dishes that participants reported with their nutrient contents using the nutrient database. For FFQ, all frequencies were standardised into ‘times per day’ by using the conversion factors (1 month = 4·3 weeks = 30·4 d). Daily intakes of energy and nutrients were estimated as follows: the standardised frequency per day × the amount of food per standard unit × their nutrient contents for each 112-food item. To calculate total intakes of energy and nutrients for each NOVA group, the amount consumed of each individual food item in the same NOVA group was summed. For example, the estimated energy intake of each 32-food item classified into UPF was then summed as total energy intake from UPF. The nutrient database for both dietary assessment methods was the Ninth Edition of the Korean Food Composition Table of the Rural Development Administration^(23)^. Our main interests were daily intakes of energy (kcal), protein (g), fat (g), saturated fat (g), carbohydrate (g), fibre (g) and Na (mg).

### Categorisation of foods according to the NOVA classification

We used the NOVA classification system that enables us to evaluate diet according to the extent and purpose of food processing^(6)^. NOVA has four food categories according to the degree and purpose of food processing: unprocessed or MPF (group 1), PCI (group 2), PF (group 3) and UPF (group 4)^(6)^. In brief, the first group indicates natural or MPF without adding substances such as sugar, salt, oils or fats and with the rare use of additives for preservation purposes. Allowable processing limits the purpose of extending the life of unprocessed foods (chilling, freezing, drying and pasteurising) and of facilitating or diversifying food preparation (removing inedible parts, crushing, grinding, roasting and fermentation of milk to make yogurt). Fruits and vegetables, grains, nuts and seeds, tea and coffee, and eggs are included in NOVA group 1. The second group indicates the PCI that are directly obtained from the first NOVA group or from nature by processes of pressing, refining, grinding, milling and spray drying. They are mostly consumed in the presence of group 1 foods as a seasoning, and the use of additives is allowed when the purpose is to preserve the product’s original properties. Sugar, honey, salt, vinegar, vegetable oils and fats (butter, lard) are major examples of group 2 foods. The third group is PF in which substances such as sugar, salt, oils or other group 2 foods are added to group 1 foods and with rare use of additives for preservation and to resist microbial contamination. Processing for the purpose of longer durability or enhancing palatability is allowed. Canned vegetables, fruits or fish; bottled vegetables, fruits and legumes; salted or sugared nuts and seeds; smoked, salted or cured meats; fruits in syrup; cheeses and unpackaged freshly made bread are recognised as group 3 foods. The fourth group is UPF and beverages, which are defined as ‘*formulations of food substances often modified by chemical processes and then assembled into ready-to-consume hyper-palatable food and drink products using flavours, colours, emulsifiers and a myriad of other cosmetic additives*’^(6)^. This group includes bread (mass-produced packaged); biscuits; cakes; cookies; chocolate; candies; ice cream, carbonated or fruit drinks; pre-prepared dishes; sausages; hog dogs; burgers; nuggets and instant soups, noodles or dumplings. The rationale and details of NOVA classification have been described elsewhere^(2)^. In the KNHANES 2016, a total of 653 food items were reported from the 24HR, and 109 food items (except for alcoholic beverages) were available from the FFQ. The final NOVA classification of food items in the FFQ is presented in Supplemental Table 1.

An investigator (S.J) classified all food and beverage items of the FFQ in the KNHANES 2016 into one of the four food groups in NOVA, and the whole classification was thoroughly cross-checked by another investigator (JYK). If there was disagreement in classifying such items, we then checked (1) product name and manufacturer information to identify raw materials and (2) contents of Na or sugar in such items based on the food code in the nutrient database. This is because that more PF are likely to contain higher levels of Na and/or sugars. For dishes with multiple ingredients, we used the ingredient database with the use of a standard recipe of Korean dishes (provided by the Korea Centres for Disease Control and Prevention) to check the amount of each ingredient in such dish. The database provides food name, food code and amount of ingredients (g) per dish. We classified mixed dishes based on the main ingredient, which can be assessed by the proportion of the amount of the main ingredient out of the total amount of dishes. All food items from both dietary assessment methods were mutually exclusively classified into one of the four NOVA groups. NOVA group 4, UPF, was the main interest in the present study.

### Assessment of socio-demographic characteristics

To present the socio-demographic characteristics of the study participants, we included age, sex, residential area, educational level, income, occupation and marital status. Trained interviewers administered the health interview questionnaires to the participants aged 19 years and older. Information on demographics (age, sex, education, income and occupation) and the residential area of all members of the sampled household was collected from the household component in the health interview. The individual component questionnaire was used to collect marital status. Such variables were manipulated for the analyses as follows: age (continuous: years and categorical: 19–29, 30–39, 40–49, 50–59, and ≥ 60 years in the unit of years), sex (categorical: men and women), residential area (categorical: urban and rural), education level (categorical: elementary school graduate or less, middle school graduate, high school graduate, and college graduate or higher), monthly household income (categorical: ≤ 100, 100–200, 200–300, and > 300 in the unit of million Korean won), occupation (categorical: non-manual, manual and no job (including students and housewives)) and marital status (categorical: married or not).

### Statistical analysis

As the measure of the consumption from each NOVA group, we presented three different types of dietary consumption of each NOVA group, expressed as: (1) crude quantity, (2) energy-adjusted quantity and (3) relative contribution to total intakes of energy and nutrients for each NOVA group (as a percentage). For the energy-adjusted quantity, six selected nutrients were adjusted for total energy intake using a residual method to adjust for measurement error due to underreporting and to make an isocaloric comparison^(10)^. Energy-adjusted nutrient intakes were calculated as the sum of the residuals obtained from the regression model with total energy intake (kcal) as the independent variable and absolute intake of nutrient as the dependent variable and the expected nutrients intake for a person with mean energy intake^(10)^.

All weights accounting for the complex sampling design of the KNHANES were applied to all further analyses. Nutrients were normalised by logarithmic transformation due to the skewed data distribution in most nutrients. The general characteristics of the study participants were described as the weighted means and their standard errors for continuous variables and as weighted prevalence and their se for categorical variables. We tested the mean difference in dietary contributions of each NOVA group to total intakes of energy and nutrients between the 24HR and the FFQ using a paired *t* test. The performance of the FFQ to apply the NOVA food classification was assessed by Pearson’s correlation coefficients between the 24HR and the FFQ. We also conducted a cross-classification analysis to compare the agreement of classification into quartiles between the two tools. For this cross-classification analysis, we classified dietary shares of NOVA groups to total intakes of energy and nutrients estimated by the 24HR and the FFQ into quartiles and determined the percentage of participants classified into the same, adjacent, or opposite quartiles. The opposite quartiles indicated gross misclassification, which ranked in the lowest or highest quartiles of the FFQ but in the highest or lowest quartiles of the 24HR as appropriate. As a sensitivity analysis, we repeated the main analyses by including those reported extreme energy intake (*n* 3258) (see online supplementary material, Supplemental Tables 2–5).

All analyses were performed using SAS software (Version 9.4, SAS Institute Inc.) while accounting for complex survey design effects. All tests were 2-sided, and significance was set at an *α* level of 0·05.

## Results

Table 1 presents the general characteristics of the study participants in the KNHANES 2016. This study included 3189 Korean adults aged 19–64 years (mean 41·2 years, se 0·4). In our sample, 50·8 % were women (1215 men and 1974 women), 42·3 % were older than 45 years of age, 87·8 % were urban residents, 87·2 % were at least a high school graduate, 67·4 % had higher than 300 million won of monthly household income, 34·9 % had manual jobs and 72·8 % were married.

**Table 1:** General characteristics of the study participants in KNHANES 2016 (*n* 3189)

	Percent	se
Sex, women	50·8	0·9
Age (years)	41·2	0·4
Age group		
19–29 years	22·2	1·3
30–39 years	22·9	1·3
40–49 years	24·9	1·1
50–59 years	23·4	1·1
≥60 years	6·6	0·5
Residential area		
Urban	87·8	2·2
Rural	12·2	2·2
Education		
≤Elementary school graduates	6·5	0·6
Middle school graduates	6·4	0·5
High school graduates	38·9	1·3
≥College graduates	48·3	1·5
Mean monthly household income (million won)	476	12
Monthly household income		
≤100	6·5	0·7
100–200	10·2	0·7
200–300	15·9	1·1
≥300	67·4	1·6
Occupation		
Non-manual	32·4	1·1
Manual	35·3	1·5
No job*	32·3	1·3
Married	72·0	1·4

KNHANES, Korea National Health and Nutrition Examination Survey.

Note: Values are expressed as weighted means (se) for continuous variables and as weighted percentages (se) for categorical variables.

* No job included students and housewives.

Table 2 shows the mean daily intakes of energy and selected nutrients estimated by the 24HR and the FFQ. For crude values, total intakes of energy, protein, fat, saturated fat, carbohydrate, fibre and Na in the 24HR were greater than those in the FFQ. The intakes of the aforementioned energy and nutrients from the MPF, PCI and UPF in the 24HR were higher than those in the FFQ. The intakes of energy and nutrients from PF in the FFQ were lower than those in the 24HR. We observed similar patterns in both energy-adjusted nutrients and percentage intake from each food. Irrespective of the method of dietary assessment, MPF contributed the most to daily intakes of energy and carbohydrates (24HR: energy 55·6 %, carbohydrate 60·0 % *v*. FFQ: energy 50·1 %, carbohydrate 60·8 %). The percent intakes of protein, fat, saturated fat, fibre and Na differed by dietary assessment methods. MPF contributed the most to daily intakes of protein, fat, saturated fat and fibre, and PF contributed the most to daily intakes of Na in the 24HR, whereas PF contributed the most to daily intakes of protein, fat, saturated fat, fibre and Na in the FFQ.

**Table 2:** Mean daily intakes of energy and nutrients from NOVA groups estimated from a 24HR and FFQ

	Crude				Energy-adjusted				Percentage of intake			
	24HR		FFQ		24HR		FFQ		24HR		FFQ	
	Mean	se	Mean	se	Mean	se	Mean	se	Mean	se	Mean	se
Total												
Energy (kcal)	2149·3	22·4	1855·2	14·4	–		–		–		–	
Protein (g)	78·2	1·0	64·3	0·6	73·2	0·6	60·6	0·3	–		–	
Fat (g)	51·1	0·8	41·9	0·5	46·0	0·5	38·9	0·3	–		–	
Saturated fat (g)	16·6	0·3	12·6	0·2	15·0	0·2	11·6	0·1	–		–	
Carbohydrate (g)	308·6	3·1	300·0	2·2	296·3	1·7	289·6	1·0	–		–	
Fibre (g)	26·1	0·4	19·1	0·2	25·1	0·3	18·3	0·2	–		–	
Na (mg)	3634·0	55·0	3214·4	32·5	3435·2	39·2	3040·5	19·9	–		–	
MPF												
Energy (kcal)	1146·5	13·7	906·2	8·6	–		–		55·6	0·5	50·1	0·4
Protein (g)	52·6	0·8	20·3	0·2	49·4	0·6	19·7	0·2	66·1	0·5	34·0	0·3
Fat (g)	21·9	0·5	8·7	0·2	19·3	0·4	8·2	0·1	41·7	0·6	21·4	0·3
Saturated fat (g)	7·6	0·2	3·8	0·1	6·6	0·1	3·5	0·1	43·8	0·6	29·5	0·4
Carbohydrate (g)	183·2	2·4	182·4*	1·8	179·4	2·1	178·7*	1·5	60·0	0·6	60·8*	0·4
Fibre (g)	15·9	0·3	6·9	0·1	15·4	0·3	6·7	0·1	58·5	0·6	35·5	0·4
Na (mg)	311·6	7·5	171·4	3·3	300·4	7·6	160·4	2·5	9·6	0·2	5·7	0·1
PCI												
Energy (kcal)	96·9	2·6	18·2	0·8	–		–		4·4	0·1	1·0	0·0
Protein (g)	0·0	0·0	0·0	0·0	0·0	0·0	0·0	0·0	0·0	0·0	0·0	0·0
Fat (g)	7·5	0·2	0·1	0·0	6·8	0·2	0·0	0·0	15·1	0·3	0·1	0·0
Saturated fat (g)	1·2	0·1	0·0	0·0	1·1	0·1	0·0	0·0	8·2	0·2	0·2	0·0
Carbohydrate (g)	7·3	0·3	4·5	0·2	6·9	0·2	4·7	0·2	2·4	0·1	1·6	0·1
Fibre (g)	0·0	0·0	0·0	0·0	0·0	0·0	0·0	0·0	0·0	0·0	0·0	0·0
Na (mg)	766·4	30·6	0·6	0·1	704·3	25·3	0·5	0·1	17·9	0·4	0·0	0·0
PF												
Energy (kcal)	167·0	4·6	480·3	6·3	–		–		7·9	0·2	25·3	0·2
Protein (g)	11·5	0·4	31·2	0·4	10·9	0·3	29·0	0·2	14·5	0·3	46·6	0·3
Fat (g)	6·3	0·3	20·8	0·3	6·0	0·2	19·3	0·2	13·3	0·4	49·7	0·3
Saturated fat (g)	1·7	0·1	4·6	0·1	1·6	0·1	4·3	0·0	11·3	0·4	38·0	0·3
Carbohydrate (g)	16·7	0·4	42·1	0·6	16·0	0·3	39·2	0·4	5·7	0·1	14·0	0·2
Fibre (g)	5·1	0·1	9·1	0·1	5·0	0·1	8·7	0·1	20·7	0·4	46·8	0·3
Na (mg)	1293·5	23·0	2059·4	24·6	1259·5	21·3	1962·2	17·3	37·7	0·6	64·2	0·3
UPF												
Energy (kcal)	752·1	15·6	450·5	7·0	–		–		32·8	0·5	23·6	0·3
Protein (g)	14·5	0·4	12·7	0·2	13·3	0·3	11·8	0·2	19·8	0·4	19·4*	0·2
Fat (g)	15·9	0·4	12·4	0·2	14·8	0·4	11·4	0·2	30·8	0·6	28·8	0·3
Saturated fat (g)	6·3	0·2	4·1	0·1	5·9	0·2	3·8	0·1	37·4	0·6	32·3	0·3
Carbohydrate (g)	103·0	2·5	70·9	1·1	94·7	1·9	66·1	0·8	32·4	0·6	23·6	0·3
Fibre (g)	5·3	0·2	3·2	0·1	4·8	0·1	2·9	0·0	21·3	0·5	17·7	0·3
Na (mg)	1312·2	29·3	983·1	15·1	1228·6	26·4	916·6	12·9	36·1	0·5	30·2	0·3

24HR, 24-hour dietary recall; MPF, unprocessed and minimally processed foods; PCI, processed culinary ingredients; PF, processed foods; UPF, ultra-processed food and drink products.

Note: Values are expressed as weighted means (se).

* *P* > 0·05 for the differences between the 24HR and the FFQ using a paired *t* test.

The relative validity of the FFQ in terms of Pearson’s correlation coefficients is given in Table 3. For crude intake, the Pearson’s correlation coefficients between the 24HR and the FFQ were 0·33–0·42 for total intakes, 0·13–0·36 for intakes of those from MPF, –0·03–0·04 for intakes of those from PCI, 0·15–0·30 for intakes of those from PF and 0·18–0·35 for intakes of those from UPF. For energy-adjusted intake, the Pearson’s correlation coefficients between the 24HR and the FFQ were 0·29–0·47 for total intakes, 0·08–0·39 for intakes of those from MPF, –0·02–0·07 for intakes of those from PCI, 0·11–0·29 for intakes of those from PF and 0·18–0·34 for intakes of those from UPF. For percentage intake, the Pearson’s correlation coefficients between the 24HR and the FFQ were 0·09–0·36 for the MPF contribution, –0·02–0·08 for the PCI contribution, 0·04–0·25 for the PF contribution and 0·25–0·37 for the UPF contribution. Compared with the correlation coefficients between the two methods for total intakes of energy and nutrients, intakes from PCI and PF mostly showed lower correlations (≤ 0·20), and those from MPF and UPF showed relatively moderate correlations in all three values (range of average: 0·22–0·25 for MPF; 0·26–0·30 for UPF).

**Table 3:** Comparison of Pearson’s correlation coefficients of energy and nutrient intake from NOVA groups estimated from a 24-h dietary recall and FFQ

	Pearson’s					
	Crude intake	*P*-value	Energy-adjusted intake^*^	*P*-value	Percentage of intake	*P*-value
Total						
Energy (kcal)	0·39	<0·0001	–		–	
Protein (g)	0·37	<0·0001	0·40	<0·0001	–	
Fat (g)	0·41	<0·0001	0·41	<0·0001	–	
Saturated fat (g)	0·42	<0·0001	0·41	<0·0001	–	
Carbohydrate (g)	0·37	<0·0001	0·47	<0·0001	–	
Fibre (g)	0·38	<0·0001	0·43	<0·0001	–	
Na (mg)	0·33	<0·0001	0·29	<0·0001	–	
Average	0·38		0·40			
MPF						
Energy (kcal)	0·34	<0·0001	–		0·32	<0·0001
Protein (g)	0·25	<0·0001	0·20	<0·0001	0·11	<0·0001
Fat (g)	0·18	<0·0001	0·15	<0·0001	0·16	<0·0001
Saturated fat (g)	0·21	<0·0001	0·18	<0·0001	0·16	<0·0001
Carbohydrate (g)	0·36	<0·0001	0·39	<0·0001	0·36	<0·0001
Fibre (g)	0·26	<0·0001	0·33	<0·0001	0·32	<0·0001
Na (mg)	0·13	<0·0001	0·08	0·0003	0·09	<0·0001
Average	0·25		0·22		0·22	
PCI						
Energy (kcal)	0·02	0·450	–		−0·02	0·420
Protein (g)	0·02	0·282	0·07	0·010	0·08	0·115
Fat (g)	−0·002	0·932	0·02	0·853	−0·01	0·525
Saturated fat (g)	0·01	0·823	0·03	0·452	0·02	0·598
Carbohydrate (g)	0·03	0·257	0·05	0·045	0·05	0·166
Fibre (g)	−0·03	0·205	−0·02	0·625	−0·01	0·004
Na (mg)	0·04	0·096	0·04	0·083	−0·01	0·703
Average	0·02		0·04		0·05	
PF						
Energy (kcal)	0·18	<0·0001	–		0·06	0·018
Protein (g)	0·16	<0·0001	0·11	<0·0001	0·04	0·036
Fat (g)	0·15	<0·0001	0·12	<0·0001	0·08	0·001
Saturated fat (g)	0·17	<0·0001	0·13	<0·0001	0·07	0·003
Carbohydrate (g)	0·16	<0·0001	0·11	<0·0001	0·05	0·021
Fibre (g)	0·18	<0·0001	0·23	<0·0001	0·24	<0·0001
Na (mg)	0·30	<0·0001	0·29	<0·0001	0·25	<0·0001
Average	0·18		0·17		0·11	
UPF						
Energy (kcal)	0·35	<0·0001	–		0·34	<0·0001
Protein (g)	0·28	<0·0001	0·26	<0·0001	0·25	<0·0001
Fat (g)	0·27	<0·0001	0·27	<0·0001	0·28	<0·0001
Saturated fat (g)	0·24	<0·0001	0·25	<0·0001	0·25	<0·0001
Carbohydrate (g)	0·34	<0·0001	0·34	<0·0001	0·37	<0·0001
Fibre (g)	0·18	<0·0001	0·18	<0·0001	0·35	<0·0001
Na (mg)	0·29	<0·0001	0·26	<0·0001	0·28	<0·0001
Average	0·28		0·26		0·30	

Abbreviations:MPF, unprocessed and minimally processed foods; PCI, processed culinary ingredients; PF, processed foods; UPF, ultra-processed food and drink products.

* Energy-adjusted intake was estimated using the residual method.

Table 4 presents the results of the cross-classification analysis. The average proportions of participants were classified into the same or adjacent quartiles as follows: 68·8 % (PCI) to 73·9 % (UPF) for crude intake; 64·2 % (PCI) to 71·8 % (UPF) for energy-adjusted intake and 66·9 % (PF) to 73·1 % (UPF) for percentage intake. The average proportions grossly misclassified ranged from 6·5 % to 12·1 %. Compared with the cross-classification agreement between the two methods for total intakes of energy and nutrients, UPF classifications showed the greatest agreement and the lowest misclassification. A sensitivity analysis using data including those reported extreme energy intake showed similar results to the main findings (*n* 3258) (see online supplementary material, Supplemental Tables 2–5).

**Table 4:** Proportion of agreement in quartile classification of energy and nutrient intake from NOVA groups estimated from a 24-h dietary recall and FFQ

	Crude intake			Energy-adjusted intake			Percentage of intake		
	Same	Adjacent	Opposite	Same	Adjacent	Opposite	Same	Adjacent	Opposite
Total									
energy (kcal)	32·1	40·3	7·3	–	–	–	–	–	–
Protein (g)	32·4	41·0	6·8	28·7	38·7	9·4	–	–	–
Fat (g)	33·9	41·4	5·4	34·9	38·9	7·1	–	–	–
Saturated fat (g)	35·8	40·3	5·4	34·4	39·8	6·3	–	–	–
Carbohydrate (g)	32·3	39·8	7·0	32·7	40·0	6·2	–	–	–
Fibre (g)	35·2	39·7	5·4	36·2	41·6	4·9	–	–	–
Na (mg)	33·0	38·9	7·3	28·5	39·2	8·4	–	–	–
Average	33·5	40·2	6·4	32·6	39·7	7·0	–	–	–
MPF									
Energy (kcal)	34·1	39·1	6·6	–	–	–	33·0	41·1	6·6
Protein (g)	28·7	39·5	8·8	26·1	39·1	10·6	28·7	37·3	10·8
Fat (g)	30·2	38·9	9·1	26·4	39·7	10·3	29·5	38·3	9·5
Saturated fat (g)	31·5	39·4	8·0	28·9	39·4	9·1	28·5	39·5	9·2
Carbohydrate (g)	35·6	39·5	5·8	35·1	42·6	4·9	35·1	40·4	5·5
Fibre (g)	37·0	41·8	5·1	39·5	40·7	4·2	34·0	39·5	6·9
Na (mg)	29·2	36·2	9·8	26·1	37·1	12·2	26·6	38·3	11·3
Average	32·3	39·2	7·6	30·3	39·8	8·5	30·8	39·2	8·5
PCI									
Energy (kcal)	25·8	34·5	15·4	–	–	–	24·8	34·3	16·4
Protein (g)	24·8	46·2	1·9^*^	23·9	37·6	13·9^*^	24·5	46·6	2·0^*^
Fat (g)	26·2	45·6	1·8^*^	24·9	38·2	11·7^*^	24·2	45·8	2·0^*^
Saturated fat (g)	26·0	45·1	1·8^*^	24·5	38·6	12·5^*^	24·4	45·9	2·1^*^
Carbohydrate (g)	27·2	35·1	14·9	25·7	37·0	12·6	27·2	33·6	15·9
Fibre (g)	83·7	–	16·3^†^	31·5	39·5	9·2	83·7	–	16·3^†^
Na (mg)	26·8	34·4	16·1	26·4	37·0	12·5	26·9	33·9	15·6
Average	34·4	40·2	9·7	26·2	38·0	12·1	33·7	40·0	10·0
PF									
Energy (kcal)	28·2	40·6	9·0	–	–	–	25·7	40·0	11·3
Protein (g)	27·8	39·8	9·2	26·8	37·6	10·7	25·9	37·7	11·7
Fat (g)	28·2	38·7	9·7	27·9	37·6	9·9	28·4	36·5	10·5
Saturated fat (g)	28·8	38·7	9·3	28·0	37·4	10·8	27·6	38·2	10·5
Carbohydrate (g)	28·6	39·8	9·4	27·3	38·4	10·6	27·3	40·0	10·7
Fibre (g)	33·4	38·4	6·5	33·2	38·9	7·2	31·5	38·1	7·9
Na (mg)	33·2	40·5	6·5	32·5	40·5	6·2	30·9	40·4	7·1
Average	29·7	39·5	8·5	29·3	38·4	9·2	28·2	38·7	10·0
UPF									
Energy (kcal)	33·0	41·0	6·3	–	–	–	33·7	40·5	6·1
Protein (g)	33·7	39·8	7·1	31·2	39·4	7·8	32·5	39·8	7·6
Fat (g)	38·2	38·7	5·6	33·7	40·7	6·1	32·8	39·1	7·0
Saturated fat (g)	36·0	40·0	5·8	35·0	39·0	6·7	31·3	38·9	7·4
Carbohydrate (g)	33·8	40·7	6·2	31·9	41·2	6·6	34·8	41·2	5·5
Fibre (g)	32·2	39·5	7·4	30·2	39·3	8·3	36·6	38·6	5·2
Na (mg)	31·8	38·9	7·0	31·4	38·0	8·5	32·9	38·9	7·1
Average	34·1	39·8	6·5	32·2	39·6	7·3	33·5	39·6	6·6

Abbreviations: MPF, unprocessed and minimally processed foods; PCI, processed culinary ingredients; PF, processed foods; UPF, ultra-processed food and drink products.

* Nutrients estimated from FFQ were not categorised into quartiles.

† Nutrients estimated from both 24-h recall and FFQ were categorised into two groups.

## Discussion

In this study, using a nationally representative sample of Korean adults, we evaluated the performance of the FFQ compared with the 24HR in categorising foods and beverages according to the NOVA system with a specific focus on UPF. Group-mean differences between the FFQ and the 24HR in intakes of selected nutrients except for energy and carbohydrates from UPF ranged from 2 % to 40 %, which were the smallest compared with those from MPF, PCI or PF. Similarly, average Pearson’s correlation coefficients between the FFQ and the 24HR in dietary shares of UPF were approximately 0·30 (*r* = 0·28 in crude intake; *r* = 0·26 in energy-adjusted intake; *r* = 0·30 in percent intake), which were the greatest compared with those of MPF, PCI or PF (*r* = 0·22–0·25 for MPF; *r* = 0·02–0·05 for PCI; *r* = 0·11–0·18 for PF). We found acceptable agreement between the FFQ and the 24HR in quartiles of UPF contributions to diet, which were 71·8–73·9 %.

Based on our results, the use of the FFQ in the KNHANES may not be appropriate in PCI and PF classifications. Compared with 24HR, FFQ underestimated crude, energy-adjusted and percent intakes of energy and most selected nutrients from PCI and overestimated those of energy and all selected nutrients from PF. These may be attributed to the following speculations. First, the FFQ consisted of a limited number of predefined item lists with aggregated similar foods or dishes into one item, whereas the 24HR was administered in a completely open-ended format. Thus, possible gaps may exist in the coverage of all foods or dishes consumed between the two dietary assessment methods. In this study, only two (added sugar and butter/margarine) out of 109 items in the FFQ and 37 out of 653 items in the 24HR were available for PCI; 54 items in the FFQ and 128 items in the 24HR were available for PF. Second, the FFQ usually does not collect detailed information, such as food preparation methods, ingredients used in mixed dishes or places of eating; thus, cooked foods or dishes are difficult to correctly classify into each NOVA group. Since the 54 items classified as PF were mostly cooked dishes with various raw ingredients using diverse cooking methods (boiling, steaming, marinating, stir frying, grilling, frying), such as soups, stews, stir-fried or marinated vegetables, PF classification may not be accurate. Third, such substantial differences in means or correlations between the 24HR and the FFQ may be due to differences in the purpose and usage between the two methods. The 24HR is used to estimate the absolute mean value for population or true distribution of intakes for the population if 2 or more days of recalls per individual are available (e.g. comparison of nutrients intakes with dietary recommendations)^(10)^. The FFQ is primarily used to estimate average long-term usual intake for individuals and is thus appropriate for most epidemiologic studies of diet–disease associations using correlations or relative risks based on relative rankings of dietary intakes^(10)^. Thus, it is necessary to consider the primary purpose of the dietary measure.

Importantly, we observed an average of 74 % good agreement in quartile classification of UPF contribution to diets. This result indicated that UPF classified with the FFQ may be valid for the purpose of qualitative comparisons, such as ranking into quantiles and dietary patterns. Although the FFQ is not appropriate for the estimation of crude intake per se from UPF, its future use with categorical analysis can still be encouraged. As observed in many diet–disease association studies, previous studies of UPF consumption–disease association studies^(7–9)^ have used dietary exposures as categorical, not continuous, for the following advantages. In nutritional epidemiology, the use of categorised intake can make the direct visualisation of actual counts of cases and non-cases according to intake level and compute relative risks. Additionally, it can minimise the possibility of a false linear trend because no assumptions are made about a dose–response relationship. Last, it can minimise the undue impacts of outliers^(10)^. Taken together, the FFQ in this study may be appropriate to use to classify UPF in a qualitative comparison rather than in an absolute intake estimation.

To our knowledge, this is the first study on the performance of the FFQ in UPF classification compared with that of the 24HR in adults. We used the 24HR as the referent because no true gold standard dietary assessment tool is available to date. Two prior studies with a similar approach as our study are available. In a study with an Italian adult sample, the NOVA FFQ with ninety-four items aiming to estimate both the absolute (g/d) and relative (% of g) intake of the NOVA groups was developed and validated with a 7-day weighted DR^(24)^. Given the correlation coefficients of 0·6 to 0·7 and Bland–Altman plots between the NOVA FFQ and the weighted DR, the NOVA FFQ has shown moderate to good validity. Another study with young children in New Zealand assessed the absolute (kcal/d) and relative (% of kcal) energy intake of the NOVA groups. Although a relatively low correlation coefficient (*r* = 0·3) was observed, agreements of correctly or adjacently classified into quartiles of energy intake from UPF between the two methods were acceptably high (73–74 %)^(25)^. Similarly, our data showed lower validity in the comparison of absolute intake and acceptably higher validity in the comparison of ranking of intake.

Several limitations in this study warrant attention. First, there is a possibility of misclassification of each NOVA group including UPF because the FFQ in this study was not explicitly designed to apply the NOVA system at the time of its inception. As such, UPF consumption assessed with the FFQ may be under- or overestimated, even though the FFQ was validated using DR^(20)^ and covered major foods and dishes frequently consumed by Koreans^(19)^. Second, our findings may not be applicable to the food-based FFQ because we assessed the whole process using a dish-based FFQ. However, food-based FFQ might be reasonable due to the limited use of MPF or UPF classification because most items classified as MPF or UPF in our FFQ were foods consumed independently (not as combinations or dishes) (see online supplementary material, Supplemental Table 1). Third, the applicability of our finding is limited to Korean adults aged 19–64 years. In addition, because those who participated in all study examinations but were excluded from the final analytic set were older, less likely to engage in higher education, higher income and non-manual job than study participants, interpretation should be made with caution. Studies targeting other groups with different demographic and/or socio-economic characteristics should be validated as appropriate. Fourth, although we used the single 24HR as the referent in this study, neither of the single 24HR and the FFQ are gold-standard measures of dietary intake. Multiple diet records or 24HR have been frequently used to test the validity of the FFQ, however, the collection of one or more diet records or 24HR is practically challenging in large-scale epidemiological studies^(10)^. Although the single 24HR may poorly represent usual individual intake due to day-to-day variations in the foods or nutrients consumed, it has been used as a usual method for estimating the mean intake of a population^(10)^. Future studies using more superior dietary assessment methods (e.g. multiple diet records or multiple 24HR) as the referent are warranted to examine the validity of the FFQ applying the NOVA classification system.

Despite these limitations, this study has considerable strengths. Unlike the Western diet, the Korean diet is characterised as dish based with the combination of multiple individual ingredients^(11)^. Given this, our finding is informative to general Korean adults because the performance of the FFQ in UPF classification has been tested under the nationally representative sample of Korean adults who completed both the 24HR and the FFQ. Furthermore, previous studies mostly focussed solely on energy intake (kcal) or food quantity (g) from UPF; however, we expanded the range of dietary share from energy to several major nutrients, including fat and Na.

In conclusion, the FFQ in the current form may not be encouraged for estimating absolute intake of UPF. Rather, its use in quantiles may be possible for investigating the association between UPF consumption and various disease outcomes based on an epidemiological study design. Further validation studies in other study populations and new development of an FFQ specifically designed to apply the NOVA classification system in the Asian population, especially in Korea, are needed to better capture UPF consumption and for future investigations on UPF and disease associations.

## Supplementary material

For supplementary material accompanying this paper visit https://doi.org/10.1017/S1368980022000179. click here to view supplementary material
