# Supplementary material for: Comparison of dietary share of ultra-processed foods assessed with a FFQ against a 24-h dietary recall in adults: results from KNHANES 2016
Source: Public Health Nutr. 2022 Jan 19;25(5):1166–75. doi: 10.1017/S1368980022000179 (PMC9991629; doi:10.1017/S1368980022000179)
Supplement: Supplementary file 1 [file S1368980022000179sup001.docx]

**Supplemental figure 1. Participant flow chart**

19-64 years of age in

KNHANES 2016

(*n* 4,750)

Missing survey weights

(*n* 947)

(n=17,135)

Valid survey weights

(*n* 3,803)

Incomplete 24HR or FFQ

(*n* 545)

Complete both 24HR and FFQ

(*n* 3,258)

Extreme FFQ-assessed energy intake (*n* 69)

Final analytic sample

(*n* 3,189)

**Supplemental table 1. Categorization of all food and beverage items captured by the semiquantitative food frequency questionnaire used in the KNHANES 2016 into the NOVA groups**

| Food item | NOVA group assignment |
| --- | --- |
| White rice | MPF |
| Barley, mixed grains | MPF |
| Bibimbap, fried rice | PF |
| Gimbap | PF |
| Curry with rice | UPF |
| Instant noodles | UPF |
| Hot noodles | UPF |
| Black bean sauce noodles | UPF |
| Cold noodles | UPF |
| Rice cake soup | PF |
| Dumplings | UPF |
| White breads | UPF |
| Butter, margarine | PCI |
| Jam | UPF |
| Sweet red bean bread, other breads with sweet red bean, other breads with cream | UPF |
| Castella, cake, chocolate pie | UPF |
| Pizza | UPF |
| Hamburger, sandwich | UPF |
| Rice cake, other rice cakes | UPF |
| Stir-fried rice cakes (Tteokbokki) | UPF |
| Cereals | UPF |
| Ox bone soup (Sullungtang), Gomguk/Gomtang, beef bone soup | PF |
| Pork backbone stew (Gamjatang) | PF |
| Boiled mud-fish soup (Chueotang) | PF |
| Spicy fish stew (Maeuntang) | PF |
| Seaweed soup | PF |
| Beef and radish soup, spicy beef soup (Yukgaejang), radish soup | PF |
| Dried pollock soup (Bugeoguk) | PF |
| Bean paste soup | PF |
| Bean paste stew, extra strong fermented soybean paste stew | PF |
| Kimchi stew, stir-fried kimchi | PF |
| Army soup | UPF |
| Tofu stew, spicy soft tofu stew | PF |
| Tofu, spicy braised tofu, soy sauce braised tofu, pan fried tofu | PF |
| Beans/beans cooked in soy sauce | PF |
| Fried eggs, egg roll | PF |
| Boiled eggs, steamed eggs | PF |
| Grilled pork belly | MPF |
| Boiled or steamed pork with soybean paste | PF |
| Spicy stir-fried pork, spicy pork Bulgogi, grilled pork rib, steamed pork rib | PF |
| Fried pork, pork cutlet | PF |
| Grilled beef | PF |
| Grilled marinated beef (Korean style beef Bulgogi) | PF |
| Processed meat | UPF |
| Korean blood sausage | UPF |
| Ginseng chicken soup (Samgye-tang) | PF |
| Stir-fried spicy chicken (Dak Galbi), spicy Korean chicken stew | PF |
| Fried chicken | PF |
| Grilled marinated duck | PF |
| Grilled or braised mackerel, grilled or braised pacific saury | PF |
| Grilled or braised belt fish, grilled or braised corbina | PF |
| Anchovy, stir-fried anchovy | PF |
| Squid, stir-fried or seasoned dried shredded squid, dried squid | PF |
| Marinated raw crab | PF |
| Salted shrimp, salted squid, salted clamp | PF |
| Fishcake | UPF |
| Bean sprouts (marinated, soup), mung bean sprouts (marinated) | PF |
| Spinach (marinated) | PF |
| Bellflower root (marinated) | PF |
| Zucchini (marinated, pancake) | PF |
| Stir-fried fern brake/bracken, seasoned aster, seasoned eggplant, and other seasoned vegetables | PF |
| Cucumber (marinated) | PF |
| Radish (marinated, picked, dried) | PF |
| Vegetable salad with dressing | PF |
| Seasoned green onion, seasoned chive | PF |
| Ssam (lettuce, perilla leaves, nappa cabbage, zucchini leaves), green peppers | MPF |
| Steamed broccoli, steamed cabbage | MPF |
| Garlic | MPF |
| Soybean paste mixed with red pepper paste | UPF |
| Cabbage kimchi | PF |
| Other kimchi | PF |
| Pickled vegetable (green pepper, garlic, perilla leaves, onion, radish), pickled cucumber | PF |
| Braised lotus roots, braised boiled burdock | PF |
| Korean pancake (chive, kimchi) | PF |
| Stir-fried noodles and vegetables | UPF |
| Stir-fried mushrooms | PF |
| Grilled laver, laver, marinated laver | PF |
| Seasoned sea lettuce, seaweed with vinegar seasoning | PF |
| Stir-fried seaweed stem | PF |
| Stir-fried potatoes, braised potatoes | PF |
| Steamed potatoes, grilled potatoes | MPF |
| Sweet potato (steamed or grilled) | MPF |
| Corn (steamed or grilled with salt or sugar) | PF |
| Milk | MPF |
| Yogurt (liquid) | UPF |
| Yogurt (solid) | UPF |
| Soybean milk | UPF |
| Strawberry | MPF |
| Tomato, cherry tomato | MPF |
| Oriental melon | MPF |
| Watermelon | MPF |
| Peach | MPF |
| Grape | MPF |
| Apple | MPF |
| Pear | MPF |
| Persimmon/dried persimmon | MPF |
| Tangerine | MPF |
| Banana | MPF |
| Orange | MPF |
| Kiwi | MPF |
| Coffee | MPF |
| Prim | UPF |
| Sugar | UPF |
| Green tea | MPF |
| Soft drink (coke/cider/fruit carbonate beverage) | UPF |
| Fruit juice | UPF |
| Mixed grain powder drink (misu-garu), traditional sweet Korean rice beverage | UPF |
| Snacks | UPF |
| Cookie/cracker | UPF |
| Chocolate | UPF |
| Ice cream | UPF |
| Peanuts | PF |
| Chestnuts | MPF |

Abbreviations: MPF, unprocessed or minimally processed foods; PCI, processed culinary ingredients; PF, processed foods; UPF, ultra-processed foods.

**Supplemental table 2. General characteristics of the study participants including those reported extreme caloric intake (*n* 3258)**

|  | **Percent** | **SE** |
| --- | --- | --- |
| Sex, women | 50.4 | 0.8 |
| Age (years) | 41.0 | 0.4 |
| Age group |  |  |
| 19-29 years | 22.8 | 1.3 |
| 30-39 years | 22.8 | 1.3 |
| 40-49 years | 24.7 | 1.0 |
| 50-59 years | 23.3 | 1.1 |
| ≥ 60 years | 6.4 | 0.5 |
| Residential area |  |  |
| Urban | 87.8 | 2.2 |
| Rural | 12.2 | 2.2 |
| Education |  |  |
| ≤ Elementary school graduates | 6.4 | 0.5 |
| Middle school graduates | 6.4 | 0.5 |
| High school graduates | 39.0 | 1.3 |
| ≥ College graduates | 48.2 | 1.5 |
| Mean monthly household income (million won) | 474 | 12 |
| Monthly household income |  |  |
| ≤ 100 | 6.5 | 0.7 |
| 100-200 | 10.3 | 0.8 |
| 200-300 | 16.0 | 1.0 |
| ≥ 300 | 67.2 | 1.6 |
| Occupation |  |  |
| Non-manual | 32.2 | 1.1 |
| Manual | 35.4 | 1.4 |
| No job^*^ | 32.4 | 1.2 |
| Married | 71.4 | 1.4 |

Abbreviation: KNHANES, Korea National Health and Nutrition Examination Survey.

Note: Values are expressed as weighted means (SE) for continuous variables and as weighted percentages (SE) for categorical variables.

^*^ No job included students and housewives.

**Supplemental table 3. Mean daily intakes of energy and nutrients from NOVA groups estimated from a 24-hour dietary recall and food frequency questionnaire including those reported extreme caloric intake (*n* 3258)**

|  | Crude |  |  |  | Energy-adjusted |  |  |  | Percentage of intake |  |  |  |
| --- | --- | --- | --- | --- | --- | --- | --- | --- | --- | --- | --- | --- |
|  | 24HR |  | FFQ |  | 24HR |  | FFQ |  | 24HR |  | FFQ |  |
|  | Mean | SE | Mean | SE | Mean | SE | Mean | SE | Mean | SE | Mean | SE |
| *Total* |  |  |  |  |  |  |  |  |  |  |  |  |
| Energy (kcal) | 2162.3 | 22.3 | 1893.6 | 16.4 | - |  | - |  | - |  | - |  |
| Protein (g) | 78.6 | 1.0 | 66.0 | 0.7 | 73.1 | 0.6 | 60.1 | 0.3 | - |  | - |  |
| Fat (g) | 51.6 | 0.8 | 43.3 | 0.6 | 46.1 | 0.5 | 38.5 | 0.3 | - |  | - |  |
| Saturated fat (g) | 16.8 | 0.3 | 13.0 | 0.2 | 15.1 | 0.2 | 11.5 | 0.1 | - |  | - |  |
| Carbohydrate (g) | 309.9 ^*^ | 3.1 | 304.7 ^*^ | 2.4 | 296.0 | 1.8 | 287.2 | 1.1 | - |  | - |  |
| Fibre (g) | 26.1 | 0.4 | 19.4 | 0.2 | 25.0 | 0.3 | 18.1 | 0.2 | - |  | - |  |
| Na (mg) | 3645.1 | 53.7 | 3289.0 | 36.3 | 3424.9 | 38.2 | 3015.1 | 20.0 | - |  | - |  |
| *MPF* |  |  |  |  |  |  |  |  |  |  |  |  |
| Energy (kcal) | 1148.6 | 13.3 | 915.1 | 8.9 | - |  | - |  | 55.3 | 0.5 | 49.9 | 0.4 |
| Protein (g) | 52.7 | 0.8 | 20.6 | 0.2 | 49.1 | 0.6 | 19.5 | 0.1 | 65.9 | 0.5 | 33.8 | 0.3 |
| Fat (g) | 22.0 | 0.5 | 9.0 | 0.2 | 19.3 | 0.4 | 8.1 | 0.1 | 41.6 | 0.6 | 21.5 | 0.3 |
| Saturated fat (g) | 7.6 | 0.2 | 3.9 | 0.1 | 6.6 | 0.1 | 3.5 | 0.1 | 43.7 | 0.6 | 29.5 | 0.4 |
| Carbohydrate (g) | 183.3 ^*^ | 2.4 | 183.7 ^*^ | 1.9 | 178.9 ^*^ | 2.1 | 177.0 ^*^ | 1.5 | 59.8 ^*^ | 0.6 | 60.5 ^*^ | 0.4 |
| Fibre (g) | 15.8 | 0.3 | 7.0 | 0.1 | 15.3 | 0.3 | 6.6 | 0.1 | 58.2 | 0.6 | 35.3 | 0.3 |
| Na (mg) | 311.9 | 7.4 | 176.6 | 3.4 | 298.9 | 7.5 | 159.4 | 2.5 | 9.5 | 0.2 | 5.7 | 0.1 |
| *PCI* |  |  |  |  |  |  |  |  |  |  |  |  |
| Energy (kcal) | 97.3 | 2.6 | 18.4 | 0.7 | - |  | - |  | 4.3 | 0.1 | 1.1 | 0.0 |
| Protein (g) | 0.0 | 0.0 | 0.0 | 0.0 | 0.0 | 0.0 | 0.0 | 0.0 | 0.0 | 0.0 | 0.0 | 0.0 |
| Fat (g) | 7.5 | 0.2 | 0.1 | 0.0 | 6.7 | 0.2 | 0.0 | 0.0 | 15.0 | 0.3 | 0.1 | 0.0 |
| Saturated fat (g) | 1.2 | 0.1 | 0.0 | 0.0 | 1.1 | 0.1 | 0.0 | 0.0 | 8.2 | 0.2 | 0.2 | 0.0 |
| Carbohydrate (g) | 7.3 | 0.3 | 4.6 | 0.2 | 6.8 | 0.2 | 4.7 | 0.2 | 2.4 | 0.1 | 1.6 | 0.1 |
| Fibre (g) | 0.0 | 0.0 | 0.0 | 0.0 | 0.0 | 0.0 | 0.0 | 0.0 | 0.0 | 0.0 | 0.0 | 0.0 |
| Na (mg) | 761.1 | 30.0 | 0.6 | 0.1 | 694.6 | 24.8 | 0.5 | 0.1 | 17.8 | 0.4 | 0.0 | 0.0 |
| *PF* |  |  |  |  |  |  |  |  |  |  |  |  |
| Energy (kcal) | 168.8 | 4.5 | 494.5 | 6.8 | - |  | - |  | 7.9 | 0.2 | 25.3 | 0.2 |
| Protein (g) | 11.6 | 0.4 | 32.3 | 0.4 | 11.0 | 0.3 | 28.7 | 0.2 | 14.5 | 0.3 | 46.6 | 0.3 |
| Fat (g) | 6.4 | 0.3 | 21.4 | 0.3 | 6.0 | 0.2 | 19.1 | 0.2 | 13.3 | 0.4 | 49.6 | 0.3 |
| Saturated fat (g) | 1.7 | 0.1 | 4.7 | 0.1 | 1.6 | 0.1 | 4.2 | 0.0 | 11.4 | 0.4 | 37.9 | 0.3 |
| Carbohydrate (g) | 16.8 | 0.4 | 43.2 | 0.6 | 16.0 | 0.3 | 38.8 | 0.4 | 5.7 | 0.1 | 14.1 | 0.2 |
| Fibre (g) | 5.1 | 0.1 | 9.2 | 0.1 | 5.0 | 0.1 | 8.6 | 0.1 | 20.8 | 0.4 | 46.8 | 0.3 |
| Na (mg) | 1298.2 | 22.4 | 2101.3 | 26.5 | 1258.8 | 21.0 | 1943.9 | 17.3 | 37.7 | 0.6 | 64.0 | 0.3 |
| *UPF* |  |  |  |  |  |  |  |  |  |  |  |  |
| Energy (kcal) | 761.0 | 15.6 | 465.6 | 7.6 | - |  | - |  | 33.0 | 0.5 | 23.7 | 0.3 |
| Protein (g) | 14.7 | 0.4 | 13.2 | 0.2 | 13.3 | 0.3 | 11.7 | 0.2 | 20.0 ^*^ | 0.4 | 19.5 ^*^ | 0.2 |
| Fat (g) | 16.2 | 0.4 | 12.8 | 0.2 | 14.9 | 0.4 | 11.3 | 0.2 | 31.0 | 0.6 | 28.8 | 0.3 |
| Saturated fat (g) | 6.4 | 0.2 | 4.3 | 0.1 | 6.0 | 0.2 | 3.8 | 0.1 | 37.5 | 0.6 | 32.3 | 0.3 |
| Carbohydrate (g) | 104.1 | 2.5 | 73.2 | 1.2 | 94.9 | 1.9 | 65.6 | 0.8 | 32.6 | 0.6 | 23.8 | 0.3 |
| Fibre (g) | 5.3 | 0.2 | 3.3 | 0.1 | 4.8 | 0.1 | 2.9 | 0.0 | 21.5 | 0.5 | 17.9 | 0.3 |
| Na (mg) | 1324.6 | 29.4 | 1010.4 | 15.8 | 1228.3 | 25.9 | 909.2 | 12.9 | 36.3 | 0.5 | 30.3 | 0.3 |

Abbreviations: KNHANES, Korea National Health and Nutrition Examination Survey; 24HR, 24-hour dietary recall; FFQ, food frequency questionnaire; MPF, unprocessed and minimally processed foods; PCI, processed culinary ingredients; PF, processed foods; UPF, ultra-processed food and drink products.

Note: Values are expressed as weighted means (SE).

^*^ *p*>0.05 for the differences between the 24HR and the FFQ using a paired t-test.

**Supplemental table 4. Comparison of Pearson’s correlation coefficients of energy and nutrient intake from NOVA groups estimated from a 24-hour dietary recall and food frequency questionnaire including participants reported extreme caloric intake (*n* 3258)**

|  | Pearson’s |  |  |  |  |  |
| --- | --- | --- | --- | --- | --- | --- |
|  | Crude intake | p-value | Energy-adjusted intake^*^ | p-value | Percentage of intake | p-value |
| *Total* |  |  |  |  |  |  |
| Energy (kcal) | 0.42 | <.0001 | - |  | - |  |
| Protein (g) | 0.40 | <.0001 | 0.40 | <.0001 | - |  |
| Fat (g) | 0.43 | <.0001 | 0.41 | <.0001 | - |  |
| Saturated fat (g) | 0.44 | <.0001 | 0.40 | <.0001 | - |  |
| Carbohydrate (g) | 0.41 | <.0001 | 0.46 | <.0001 | - |  |
| Fibre (g) | 0.40 | <.0001 | 0.44 | <.0001 | - |  |
| Na (mg) | 0.35 | <.0001 | 0.30 | <.0001 | - |  |
| **Average** | **0.41** |  | **0.40** |  |  |  |
| *MPF* |  |  |  |  |  |  |
| Energy (kcal) | 0.40 | <.0001 | - |  | 0.33 | <.0001 |
| Protein (g) | 0.30 | <.0001 | 0.25 | <.0001 | 0.12 | <.0001 |
| Fat (g) | 0.20 | <.0001 | 0.15 | <.0001 | 0.15 | <.0001 |
| Saturated fat (g) | 0.23 | <.0001 | 0.18 | <.0001 | 0.16 | <.0001 |
| Carbohydrate (g) | 0.43 | <.0001 | 0.44 | <.0001 | 0.37 | <.0001 |
| Fibre (g) | 0.48 | <.0001 | 0.48 | <.0001 | 0.33 | <.0001 |
| Na (mg) | 0.16 | <.0001 | 0.10 | 0.0003 | 0.09 | <.0001 |
| **Average** | **0.31** |  | **0.27** |  | **0.22** |  |
| *PCI* |  |  |  |  |  |  |
| Energy (kcal) | 0.03 | 0.2652 | - |  | -0.02 | 0.4707 |
| Protein (g) | 0.03 | 0.1815 | 0.07 | 0.0049 | 0.08 | 0.1099 |
| Fat (g) | 0.003 | 0.9059 | 0.02 | 0.4997 | -0.01 | 0.5577 |
| Saturated fat (g) | 0.01 | 0.6678 | 0.03 | 0.2414 | 0.02 | 0.5771 |
| Carbohydrate (g) | 0.03 | 0.1586 | 0.05 | 0.0417 | 0.05 | 0.1745 |
| Fibre (g) | -0.03 | 0.1543 | -0.02 | 0.5524 | -0.01 | 0.0039 |
| Na (mg) | 0.04 | 0.0593 | 0.05 | 0.0589 | -0.0047 | 0.8227 |
| **Average** | **0.02** |  | **0.04** |  | **0.05** |  |
| *PF* |  |  |  |  |  |  |
| Energy (kcal) | 0.21 | <.0001 | - |  | 0.06 | 0.0062 |
| Protein (g) | 0.18 | <.0001 | 0.11 | <.0001 | 0.05 | 0.0159 |
| Fat (g) | 0.17 | <.0001 | 0.13 | <.0001 | 0.09 | 0.0002 |
| Saturated fat (g) | 0.18 | <.0001 | 0.13 | <.0001 | 0.07 | 0.0019 |
| Carbohydrate (g) | 0.18 | <.0001 | 0.11 | <.0001 | 0.06 | 0.0128 |
| Fibre (g) | 0.19 | <.0001 | 0.24 | <.0001 | 0.24 | <.0001 |
| Na (mg) | 0.31 | <.0001 | 0.30 | <.0001 | 0.26 | <.0001 |
| **Average** | **0.20** |  | **0.17** |  | **0.12** |  |
| *UPF* |  |  |  |  |  |  |
| Energy (kcal) | 0.37 | <.0001 | - |  | 0.35 | <.0001 |
| Protein (g) | 0.32 | <.0001 | 0.28 | <.0001 | 0.26 | <.0001 |
| Fat (g) | 0.29 | <.0001 | 0.28 | <.0001 | 0.28 | <.0001 |
| Saturated fat (g) | 0.26 | <.0001 | 0.26 | <.0001 | 0.25 | <.0001 |
| Carbohydrate (g) | 0.36 | <.0001 | 0.35 | <.0001 | 0.38 | <.0001 |
| Fibre (g) | 0.22 | <.0001 | 0.21 | <.0001 | 0.37 | <.0001 |
| Na (mg) | 0.33 | <.0001 | 0.29 | <.0001 | 0.29 | <.0001 |
| **Average** | **0.31** |  | **0.28** |  | **0.31** |  |

Abbreviations: KNHANES, Korea National Health and Nutrition Examination Survey; FFQ, food frequency questionnaire; MPF, unprocessed and minimally processed foods; PCI, processed culinary ingredients; PF, processed foods; UPF, ultra-processed food and drink products.

^*^ Energy-adjusted intake was estimated using the residual method.

**Supplemental table 5. Proportion of agreement in quartile classification of energy and nutrient intake from NOVA groups estimated from a 24-hour dietary recall and food frequency questionnaire including participants reported extreme caloric intake (*n* 3258)**

|  | Crude intake |  |  | Energy-adjusted intake |  |  | Percentage of intake |  |  |
| --- | --- | --- | --- | --- | --- | --- | --- | --- | --- |
|  | Same | Adjacent | Opposite | Same | Adjacent | Opposite | Same | Adjacent | Opposite |
| *Total* |  |  |  |  |  |  |  |  |  |
| Energy (kcal) | 32.9 | 40.3 | 7.0 | - | - | - | - | - | - |
| Protein (g) | 32.8 | 41.0 | 6.3 | 28.7 | 39.0 | 9.4 | - | - | - |
| Fat (g) | 34.7 | 41.5 | 5.1 | 34.0 | 39.0 | 7.3 | - | - | - |
| Saturated fat (g) | 36.2 | 40.5 | 5.2 | 34.5 | 39.2 | 6.6 | - | - | - |
| Carbohydrate (g) | 32.9 | 39.6 | 6.8 | 32.9 | 39.4 | 6.6 | - | - | - |
| Fibre (g) | 35.4 | 39.7 | 5.5 | 36.4 | 41.2 | 4.9 | - | - | - |
| Na (mg) | 33.3 | 39.0 | 7.3 | 29.0 | 38.9 | 8.4 | - | - | - |
| **Average** | **34.0** | **40.2** | **6.2** | **32.6** | **39.4** | **7.2** | **-** | **-** | **-** |
| *MPF* |  |  |  |  |  |  |  |  |  |
| Energy (kcal) | 34.4 | 38.9 | 6.5 | - | - | - | 33.1 | 41.3 | 6.5 |
| Protein (g) | 29.2 | 39.3 | 8.7 | 26.2 | 39.0 | 10.4 | 29.0 | 37.4 | 10.8 |
| Fat (g) | 31.1 | 38.5 | 9.0 | 26.5 | 39.4 | 10.2 | 29.4 | 38.3 | 9.5 |
| Saturated fat (g) | 32.0 | 39.2 | 8.1 | 28.9 | 39.3 | 9.0 | 28.4 | 39.8 | 9.3 |
| Carbohydrate (g) | 35.6 | 39.2 | 5.8 | 35.3 | 42.2 | 5.1 | 35.0 | 41.0 | 5.3 |
| Fibre (g) | 37.1 | 41.5 | 5.3 | 39.5 | 41.0 | 4.1 | 33.8 | 39.9 | 6.8 |
| Na (mg) | 29.2 | 36.4 | 9.5 | 26.1 | 37.6 | 11.9 | 26.7 | 38.6 | 11.0 |
| **Average** | **32.7** | **39.0** | **7.6** | **30.4** | **39.8** | **8.4** | **30.7** | **39.5** | **8.5** |
| *PCI* |  |  |  |  |  |  |  |  |  |
| Energy (kcal) | 26.3 | 34.5 | 15.1 | - | - | - | 25.0 | 34.2 | 16.2 |
| Protein (g) | 25.0 | 46.2 | 1.9^*^ | 24.0 | 37.7 | 13.8^*^ | 24.7 | 46.7 | 1.9^*^ |
| Fat (g) | 26.4 | 45.6 | 1.9^*^ | 24.8 | 38.6 | 11.6^*^ | 24.1 | 45.9 | 2.1^*^ |
| Saturated fat (g) | 26.1 | 45.1 | 1.8^*^ | 24.5 | 38.9 | 12.4^*^ | 24.3 | 46.0 | 2.1^*^ |
| Carbohydrate (g) | 27.0 | 35.3 | 14.8 | 25.7 | 36.8 | 12.5 | 27.2 | 33.5 | 15.7 |
| Fibre (g) | 83.6 | - | 16.4^†^ | 31.7 | 39.8 | 9.2 | 83.6 | - | 16.4^†^ |
| Na (mg) | 27.0 | 34.2 | 15.8 | 26.2 | 37.4 | 12.5 | 26.9 | 34.0 | 15.5 |
| **Average** | **34.5** | **40.1** | **9.7** | **26.1** | **38.2** | **12.0** | **33.7** | **40.0** | **10.0** |
| *PF* |  |  |  |  |  |  |  |  |  |
| Energy (kcal) | 28.7 | 40.2 | 8.9 | - | - | - | 26.2 | 39.9 | 11.2 |
| Protein (g) | 28.3 | 39.7 | 9.2 | 27.4 | 37.2 | 10.7 | 25.9 | 38.1 | 11.5 |
| Fat (g) | 29.0 | 38.4 | 9.6 | 27.9 | 37.6 | 10.2 | 28.1 | 36.8 | 10.4 |
| Saturated fat (g) | 29.2 | 38.4 | 9.3 | 28.6 | 36.7 | 11.0 | 28.0 | 38.1 | 10.3 |
| Carbohydrate (g) | 29.0 | 39.7 | 9.5 | 27.3 | 38.7 | 10.5 | 27.4 | 39.9 | 10.4 |
| Fibre (g) | 33.8 | 38.3 | 6.6 | 33.0 | 39.1 | 7.2 | 31.2 | 38.5 | 7.9 |
| Na (mg) | 33.7 | 40.3 | 6.6 | 32.6 | 40.4 | 6.3 | 31.1 | 40.7 | 6.9 |
| **Average** | **30.2** | **39.3** | **8.5** | **29.5** | **38.3** | **9.3** | **28.3** | **38.9** | **9.8** |
| *UPF* |  |  |  |  |  |  |  |  |  |
| Energy (kcal) | 33.2 | 41.0 | 6.2 | - | - | - | 33.8 | 40.4 | 6.1 |
| Protein (g) | 33.6 | 40.2 | 7.1 | 30.9 | 39.4 | 8.0 | 32.2 | 40.1 | 7.7 |
| Fat (g) | 38.4 | 38.8 | 5.5 | 33.7 | 40.4 | 6.2 | 33.0 | 38.9 | 6.9 |
| Saturated fat (g) | 36.1 | 40.0 | 5.6 | 35.1 | 38.7 | 6.7 | 31.3 | 38.9 | 7.4 |
| Carbohydrate (g) | 34.1 | 40.6 | 6.0 | 32.4 | 40.8 | 6.8 | 34.9 | 41.2 | 5.6 |
| Fibre (g) | 32.5 | 39.5 | 7.4 | 29.9 | 39.4 | 8.5 | 37.1 | 38.2 | 5.2 |
| Na (mg) | 32.3 | 39.1 | 6.9 | 31.3 | 38.0 | 8.5 | 32.8 | 39.2 | 6.9 |
| **Average** | **34.3** | **39.9** | **6.4** | **32.2** | **39.4** | **7.4** | **33.6** | **39.5** | **6.6** |

Abbreviations: KNHANES, Korea National Health and Nutrition Examination Survey; FFQ, food frequency questionnaire; MPF, unprocessed and minimally processed foods; PCI, processed culinary ingredients; PF, processed foods; UPF, ultra-processed food and drink products.

^*^ Nutrients estimated from FFQ were not categorized into quartiles.

^†^ Nutrients estimated from both 24-hr recall and FFQ were categorized into two groups.
